# TP53/BRAF mutation as an aid in predicting response to immune-checkpoint inhibitor across multiple cancer types

**DOI:** 10.18632/aging.203980

**Published:** 2022-03-27

**Authors:** Jia-Zheng Cao, Gao-Sheng Yao, Fei Liu, Yi-Ming Tang, Peng-Ju Li, Zi-Hao Feng, Jun-Hang Luo, Jin-Huan Wei

**Affiliations:** 1 Department of Urology, Jiangmen Central Hospital, Jiangmen, Guangdong, China; 2 Department of Urology, The First Affiliated Hospital of Sun Yat-Sen University, Guangzhou, Guangdong, China; 3 Department of Urology, National Cancer Center, Cancer Hospital, Chinese Academy of Medical Sciences and Peking Union Medical College, Beijing, China

**Keywords:** immune-checkpoint inhibitor therapies, TP53, BRAF, prognosis, precision medicine

## Abstract

Immunotherapy with checkpoint inhibitors, such as PD-1/PD-L1 blockage, is becoming standard of practice for an increasing number of cancer types. However, the response rate is only 10%-40%. Thus, identifying biomarkers that could accurately predict the ICI-therapy response is critically important. We downloaded somatic mutation data for 46,697 patients and tumor-infiltrating immune cells levels data for 11070 patients, then combined TP53 and BRAF mutation status into a biomarker model and found that the predict ability of TP53/BRAF mutation model is more powerful than some past models. Commonly, patients with high-TMB status have better response to ICI therapy than patients with low-TMB status. However, the genotype of TP53^MUT^BRAF^WT^ in high-TMB status cohort have poorer response to ICI therapy than the genotype of BRAF^MUT^TP53^WT^ in low-TMB status (Median, 18 months vs 47 month). Thus, TP53/BRAF mutation model can add predictive value to TMB in identifying patients who benefited from ICI treatment, which can enable more informed treatment decisions.

## INTRODUCTION

Immune-checkpoint inhibitor (ICI) therapies have shown unprecedented durable responses in patients with advanced-stage cancers, including the success of anti-programmed cell death protein 1 (PD-1), anti-programmed death-ligand 1 (PD-L1) and anti-cytotoxic T-lymphocyte-associated protein 4 (CTLA-4), but the response rate is only 10%-40% [1, 2]. Therefore, it is important to identify the biomarkers that can accurately predict the ICI-therapy response.

More and more studies showed tumor mutation burden (TMB) is a clinical useful biomarker for identifying patients who benefited from ICI treatment [3, 4]. Recently, a pan-cancer study showed combining POLE and POLD1 mutation status into a simple model also can efficiently predict response to ICI therapy [5]. TP53 is one of the most frequently mutated gene in human cancers and has been formulated in a large number of studies for functions and mechanisms [6]. In brief, wild-type p53 plays a vital role in maintaining genomic stability and preventing oncogenesis by regulating many cellular processes, including promoting cell growth arrest, DNA repair, modulating autophagy and cancer metabolism [7], and TP53 is highly mutated in about 50% of human cancers. BRAF, is located on human chromosome 7 and encodes a RAS-regulated serine-threonine kinase that plays a part in ERK/MAPK signaling pathway. At the same time, the pathway is not only involved in regulating cellular biological functions, but is also related to tumor formation [8]. Up to this day, mutations in BRAF have been reported extensively in a variety of benign and malignant tumors [9, 10]. Comparing with POLE and POLD1, the mutation of TP53 and BRAF are more common in human cancer, and TP53 and BRAF had been shown to be linked to ICI therapies responses [11, 12].

In this study, we combined TP53 and BRAF mutation status into a biomarker model and found that the predict ability of TP53/BRAF mutation model is more powerful than POLE/POLD1 mutation model, and the combination of TP53/BRAF mutation model and TMB can more accurately predict the response to ICI therapy. Furthermore, we propose several possible molecular signaling pathways for the effect of TP53/BRAF mutations on the predictive value of ICI treatment response.

## MATERIALS AND METHODS

In this study, somatic mutation data for 46,697 patients were downloaded from cBioPortal (https://www.cbioportal.org) [13]. All nonsynonymous mutations were taken into account. The overall survival (OS) of 1,661 patients who received ICI therapy was defined from the date of the first ICI treatment to the time of death or most recent follow-up, and TMB was defined as the total number of somatic nonsynonymous mutations normalized to the total number of megabases sequenced [14].

The data of tumor-infiltrating immune cells levels for 11070 patients from TCGA by CIBERSORT^14^ was download from Tumor Immune Estimation Resource (TIMER) version 2.0 [15] (http://timer.cistrome.org/infiltration_estimation_for_tcga.csv.gz). The expression profiles of mRNAs and clinical survival data of 33 tumor types were obtained from the Pan-Cancer Atlas (https://gdc.cancer.gov/about-data/publications/pancanatlas).

The limma package V3.34.9 in R was used to identify differentially expressed mRNAs. Gene Ontology (GO) and Kyoto Encyclopedia of Genes and Genomes (KEGG) analyses were identified and visualized using R packages “clusterProfiler”. The cBioPortal online analysis tool was used for mutual exclusivity analysis between TP53 mutation and BRAF mutation. For survival analysis, Kaplan-Meier survival curves were generated and compared using the log-rank test, and the Cox regression model was used for multivariate survival analysis. Statistical tests were done with R software (version 4.0.2). Statistical significance was set at p values less than 0.05. Ethical approval was waived because we used only publicly available data and materials in this study.

### Availability of data and materials

The datasets presented in this study can be found in online repositories. The names of the repositories and accession numbers can be found in the article material.

## RESULTS

### TP53/BRAF mutation model has high frequency

**Figure 1:**
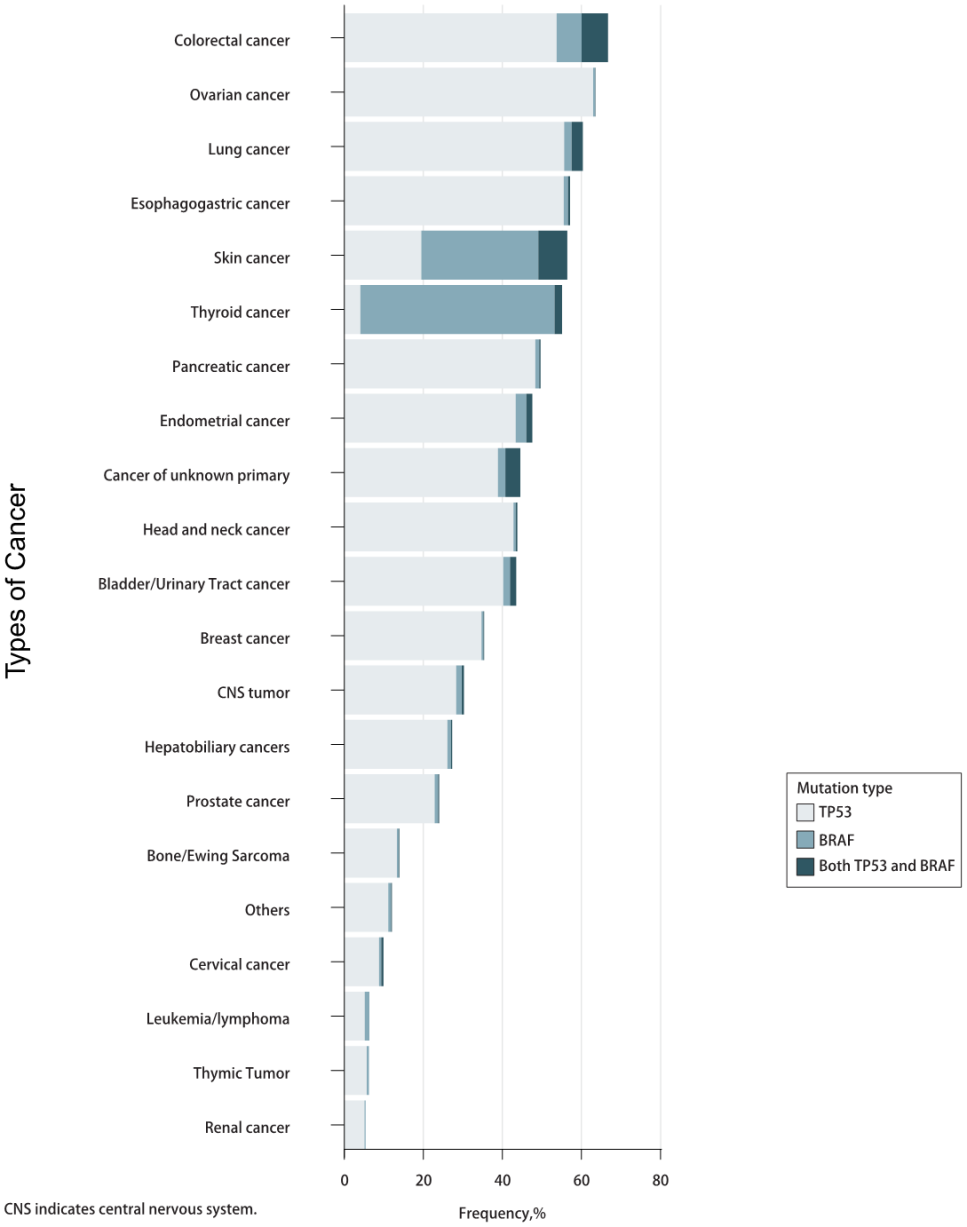
Prevalence of TP53/BRAF mutations in pan-cancer.

The prevalence of TP53 and BRAF mutations in 46,697 patients with different cancer types is summarized in Figure 1. The mutation frequencies of TP53 and BRAF (33.51% and 5.30%) were significantly higher than that of POLE and POLD1 (2.74% and 1.45%). The relationships between TP53 mutation and BRAF mutation are mutually exclusive (Table 1).

**Table 1:** Mutual exclusivity analysis between TP53 mutation and BRAF mutation in the whole cohort, TCGA subset, and MSKCC subset.

**Cohorts**	**TP53^WT^** **BRAF^WT^**	**TP53^MUT^** **BRAF^WT^**	**BRAF^MUT^** **TP53^WT^**	**TP53^MUT^** **BRAF^MUT^**	**Log2 odds ratio**	**p-value**	**q-value**
Whole cohort	27745	13519	1642	646	-0.309	<0.001	<0.001
TCGA subset	5683	3661	636	209	-0.971	<0.001	<0.001
MSKCC subset	798	691	119	53	-0.959	<0.001	<0.001

TCGA, The Cancer Genome Atlas; MSKCC, Memorial Sloan Kettering Cancer Center.

### TP53/BRAF mutation model can predict immunotherapeutic effect and prognosis of patients

**Figure 2:**
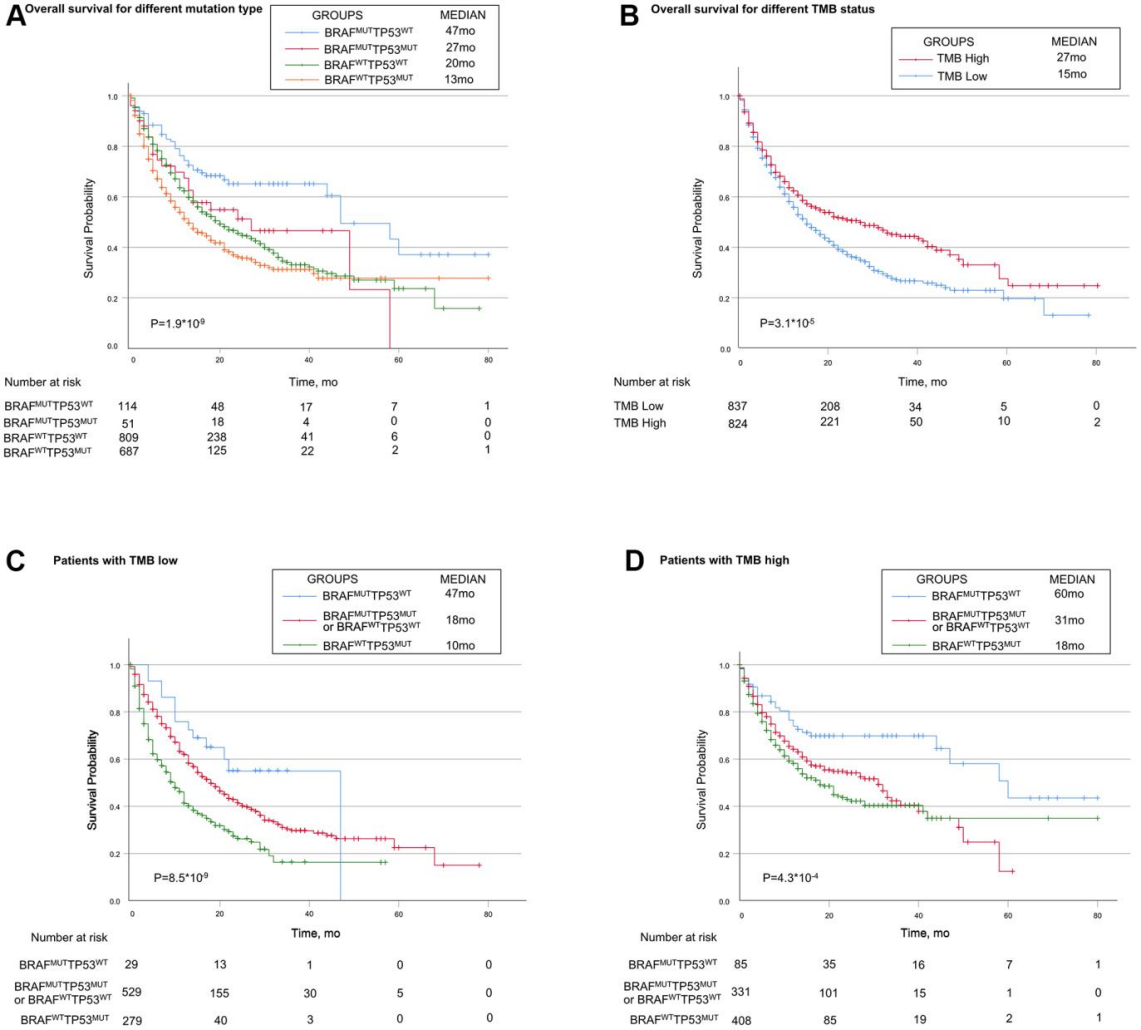
**Associations of TP53 and BRAF mutation types with prognosis in patients treated with immune checkpoint inhibitors.** (**A**) Patients with the BRAF mutation alone had the best prognosis, while patients with TP53 mutation alone had the worst prognosis. Patients with mutations in both or none had median survival. (**B**) Patients in high-TMB status group had longer OS than patients in low-TMB status group. (**C**, **D**) In both high-TMB/low-TMB status groups, TP53^MUT^BRAF^WT^ indicated poorer OS, while BRAF^MUT^TP53^WT^ did the opposite. BRAF indicates B-Raf Proto-Oncogene, Serine/Threonine Kinase gene; TP53 indicates tumor protein p53 gene; MUT indicates mutant genes; WT indicates wild type genes; TMB indicates tumor mutation burden; MSI indicates microsatellite instable.

Based on the mutually exclusive relationship between TP53 mutation and BRAF mutation, we explored the immunotherapy response in patients with different combinations of TP53 mutation and BRAF mutation. Patients were divided into four genotypes, patients with BRAF mutation alone (BRAF^MUT^TP53^WT^) showed favorable survival (Median, 47 months), while those with TP53 mutation alone (TP53^MUT^BRAF^WT^) had the worst survival (Median, 13 months). Patients with both mutations or neither mutation (TP53^MUT^BRAF^MUT^ or TP53^WT^BRAF^WT^) showed moderate survival (Median, 27 months and 20 months, respectively) (Figure 2A).

In multivariable Cox regression analysis, TP53/BRAF mutation model and TMB were independent predictive factors for identifying patients who benefited from ICI treatment (both *P*<0.0001). However, POLE/POLD1 mutation model and MSI were not independent predictive factors (both *P*>0.05) (Table 2).

**Table 2:** Univariate and multivariable association of the TP53/BRAF mutation model with overall survival in 1,661 patients who received ICI therapy.

**Parameters**	**Univariate**			**Multivariable**	
	**HR (95%CI)**	**p value**		**HR (95%CI)**	**p value**
Gender	0.88 (0.77-1.01)	0.078		0.89 (0.77-1.02)	0.09
Age	1.00 (0.99-1.00)	0.071		1.00 (0.99-1.00)	0.449
POLE/POLD1 mutation model	0.62 (0.45-0.84)	0.002		0.87 (0.63-1.21)	0.399
TMB	0.98 (0.98-0.99)	<0.0001		0.98 (0.97-0.99)	<0.0001
MSI	0.98 (0.97-1.00)	0.044		1.01 (0.99-1.03)	0.235
Cancer type	0.95 (0.93-0.98)	<0.0001		0.96 (0.94-0.98)	0.0003
TP53/BRAF mutation model	1.41 (1.26-1.58)	<0.0001		1.42 (1.26-1.60)	<0.0001

TMB, tumor mutation burden; MSI, microsatellite instable.

Patients in high-TMB status group (the median TMB as cutoff) had longer OS than patients in low-TMB status group (median, 27 months vs 15 month; *P* =0.000031, Figure 2B). When stratified by TMB status, TP53/BRAF mutation model was still a statistically significant model for predicting ICI-therapy response. In both high-TMB status group and low-TMB status group, TP53/BRAF mutation model can successfully divide patients into three risk stratification: good response genotype (BRAF^MUT^TP53^WT^), intermediate response genotype (TP53^MUT^BRAF^MUT^ or TP53^WT^BRAF^WT^), and poor response genotype (TP53^MUT^BRAF^WT^) (Figure 2C, 2D). In addition, the TP53/BRAF mutation model remained a statistically significant model when stratified according to patients' clinical information. Regardless of gender or age, TP53/BRAF mutation model can still classify patients into three risk stratification (Supplementary Figure 1A–1D).

We further performed MSI analysis and found that the low-MSI status group had a better prognosis (median, 19 months vs 15 month; *P*=0.0095, Supplementary Figure 1E). Exactly like the TMB model, MSI status can also stratify patients with mutated genetic risk (Supplementary Figure 1F, 1G).

### TP53/BRAF mutation is an immune-related model

**Figure 3:**
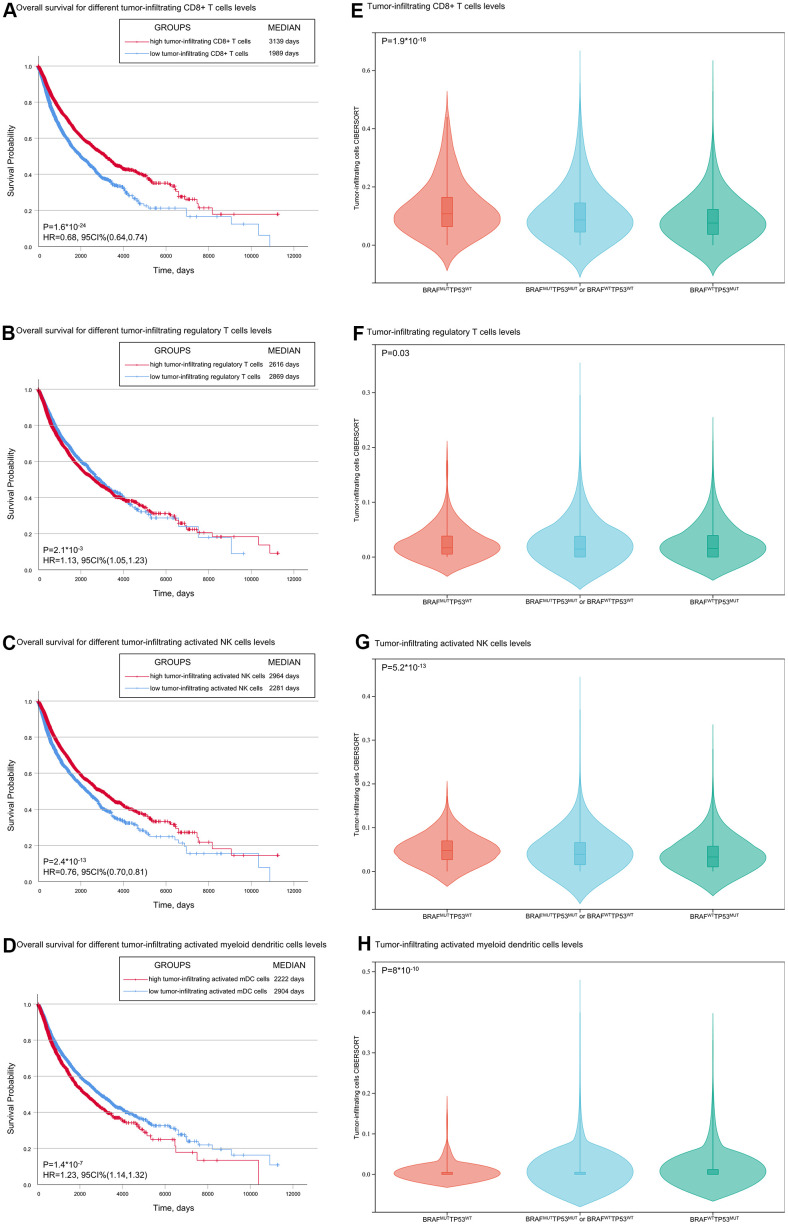
**Associations of overall survival and TP53 and BRAF mutation types with tumor-infiltrating immune cells.** (**A**, **B**) Patients with low tumor-infiltrating CD8+ T cells/activated NK cells had shorter OS than patients with high tumor-infiltrating CD8+ T cells/activated NK cells. (**C**, **D**) Patients with low tumor-infiltrating regulatory T cells /activated myeloid dendritic cells had shorter OS than patients with high regulatory T cells /activated myeloid dendritic cells. (**E**–**H**) The level of tumor infiltrating CD8+ T cells was correlated with the mutation of TP53/BRAF. Data was from TCGA database.

**Figure 4:**
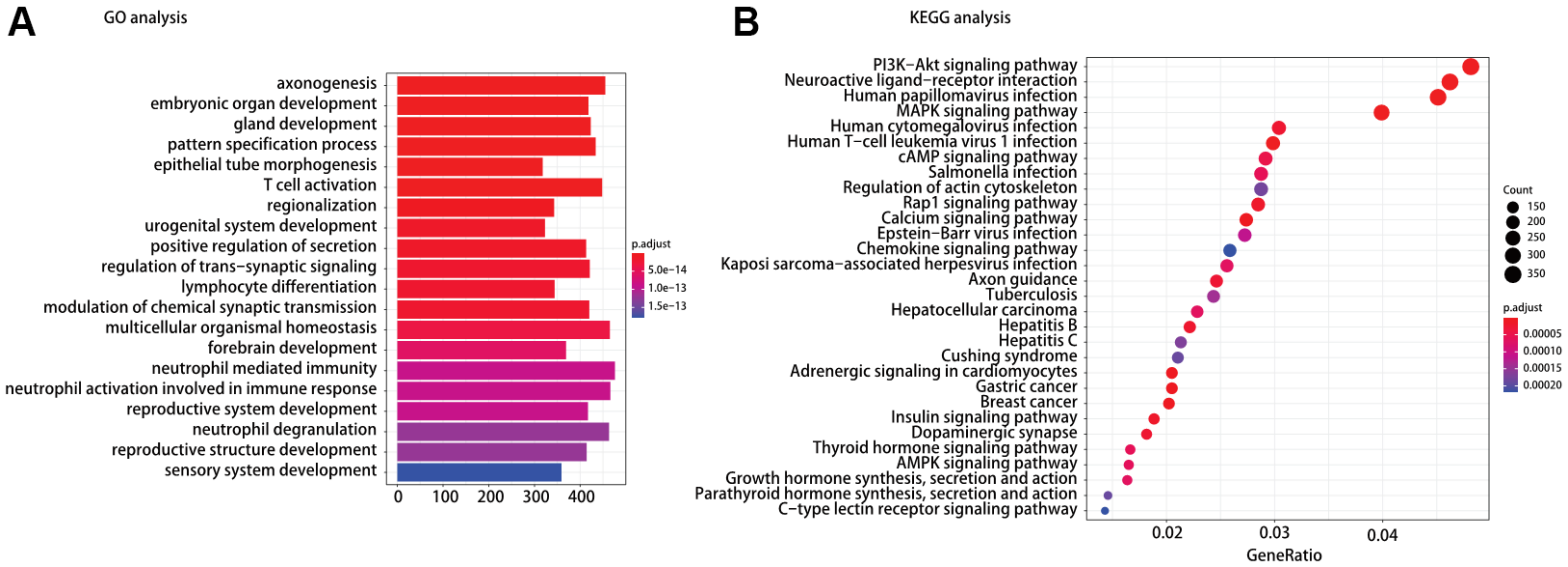
(**A**) Gene ontology (GO) and (**B**) Kyoto encyclopedia of gene and genomes (KEGG) pathway analysis of different expression mRNAs between TP53^MUT^BRAF^WT^ and BRAF^MUT^TP53^WT^.

It is generally admitted that CD8+ T cells are directly involved in antitumor cytotoxic responses, and accumulating evidence indicates that tumor-infiltrating CD8+ T cells predict the efficacy of ICI therapy [16–18]. The data from TCGA showed that patients with high tumor-infiltrating CD8+ T cells had longer OS than patients with low tumor-infiltrating CD8+ T cells (Figure 3A). We investigated whether TP53/BRAF mutation was correlated with the level of tumor-infiltrating CD8+ T cells. Patients with BRAF^MUT^TP53^WT^ showed the highest level of tumor-infiltrating CD8+ T cells, patients with TP53^MUT^BRAF^MUT^ or TP53^WT^BRAF^WT^ showed moderate level of tumor-infiltrating CD8+ T cells, and patients with TP53^MUT^BRAF^WT^ showed the lowest level of CD8+ T cells (Figure 3E). In addition, we further analyzed the correlation between other typical tumor-infiltrating immune cells and patient outcomes (Figure 4B–4D), as well as TP53/BRAF mutation (Figure 3F–3H).

In addition to tumor-infiltrating immune cells, we also analyzed the relationship between TP53/BRAF mutation model and other immune-related genes. Several representative genes were selected, such as immune-suppress genes, like S100A8 and S100A9 in myeloid-derived suppressor cells (MDSC), LRP1 in Regulatory T cells; major histocompatibility complex (MHC) related genes (HlA.DPA1, HlA.DPB1); and immune checkpoints related gene PDCD1. And The violin diagrams about the relative expression quantity of each group was drawn (Supplementary Figure 2).

### Mechanism prediction of TP53/BRAF mutation model

To understand the mechanism of oncogenesis underlying TP53/BRAF mutation correlates with response to ICI, functional enrichment characterization of different expression mRNAs between TP53^MUT^BRAF^WT^ and BRAF^MUT^TP53^WT^ was performed by GO and KEGG analysis. According to GO analysis, we found that the enriched GO terms were including T cell activation and lymphocyte differentiation. Moreover, KEGG pathway analysis indicated that most of different expression mRNAs were involved in PI3K−Akt signaling pathway, MAPK signaling pathway, Rap1 signaling pathway, chemokine signaling pathway, and AMPK signaling pathway in cancer (Figure 4).

## DISCUSSION

Up to this day, ICI therapies have shown powerful responses in cancer patients. However, the rate is not ideal enough, and the methods have the potential to play a greater role in the clinic. It’s critical to build more effective biomarker models and stratify the patients for predicting prognosis and applying better individualized treatments.

In the POLE/POLD1 mutation model, patients with either POLE or POLD1 mutations was associated with better ICI therapy response and longer OS than the wild-type population (34months vs 18months) [5]. However, the POLE/POLD1 mutation model was not a significant predictive factor for ICI therapy response after multivariable adjustment of TMB and TP53/BRAF mutation model. TP53/BRAF mutation model was a powerful and independent predictive factor for identifying patients who benefited from ICI treatment. In advanced tumors, TP53 and BRAF mutations are more common than POLE and POLD1 mutations, and TP53/BRAF mutation model is better than POLE/POLD1 mutation model in predicting ICI treatment response.

The biological implications of a mutually exclusive TP53 mutation and BRAF mutation are not understood at present. As mentioned above, TP53 is a tumor suppressor gene involved in the regulation of cell growth [19], BRAF is an oncogene involved in cellular responses to growth signals [20]. Missense mutations, insertions or deletions of TP53 lead to TP53 inactivation are very common. BRAF mutations, such as BRAF V600E mutations, cause the continuous activation of the downstream MEK-ERK signaling pathway [21]. In this study, concurrent TP53 mutation and BRAF mutation was seen in a small number of patients. Tumors carrying both TP53 mutations and BRAF mutations are less likely to response to ICI therapy than those showing only BRAF mutation. This could account for the TP53 inactivation and BRAF activation might be genetically redundant, and that alteration in both genes does not confer a further advantage.

The molecular mechanisms explaining the effects of TP53/BRAF mutation on predictive value for ICI therapy response are presently unknown. Previously, we have shown statistically that the level of tumor-infiltrating CD8+ T cells is correlated with TP53/BRAF mutations, which may be one of the causes. According to KEGG analysis, we found five enriched signaling pathways closely related to tumor immunity. The PI3K−Akt signaling pathway plays a critical role in T and B cell development [22, 23]. The BRAF-MAPK signaling pathway correlates with the production of various immunosuppressive factors in regulating cancer-immune evasion [24]. The Rap1 signaling pathway activation leads to increased integrin affinity, leukocytes arrest rolling and actively lymphocyte migration and adhesion [25–27]. Chemokines signaling pathway are key molecules involved in the migration and homeostasis of immune cells [28]. The AMPK signaling pathway is involved in shaping the activity of lymphocytes [29, 30]. The above pathways may explain potential reasons why TP53/BRAF mutation of cancer patients contributes to the ICI therapy response. More detailed and specific studies are needed to elucidate the precise molecular mechanisms.

In this study, we show that a novel TP53/BRAF mutation model provides significant information about the stratification of response to ICI-therapy. Commonly, patients with high-TMB status have better response to ICI therapy than patients with low-TMB status. However, the genotype of TP53^MUT^BRAF^WT^ in high-TMB status cohort have poorer response to ICI therapy than the genotype of BRAF^MUT^TP53^WT^ in low-TMB status (Median, 18 months vs 47 month). Thus, TP53/BRAF mutation model can add predictive value to TMB in identifying patients who benefited from ICI treatment, which can enable more informed treatment decisions.

## CONCLUSIONS

we combined TP53 and BRAF mutation status into a biomarker model which owns the ability to be more efficient than the POLE/POLD1 mutation model, and the combination of TP53/BRAF mutation model and TMB can more accurately predict the response to ICI therapy.

## Supplementary Material

Supplementary Figures
