# Supplementary material for: TP53/BRAF mutation as an aid in predicting response to immune-checkpoint inhibitor across multiple cancer types
Source: Aging (Albany NY). 2022 Mar 27;14(6):2868–79. doi: 10.18632/aging.203980 (PMC9004558; doi:10.18632/aging.203980)
Supplement: Supplementary Figures [file aging-14-203980-s001.pdf]

SUPPLEMENTARY FIGURES

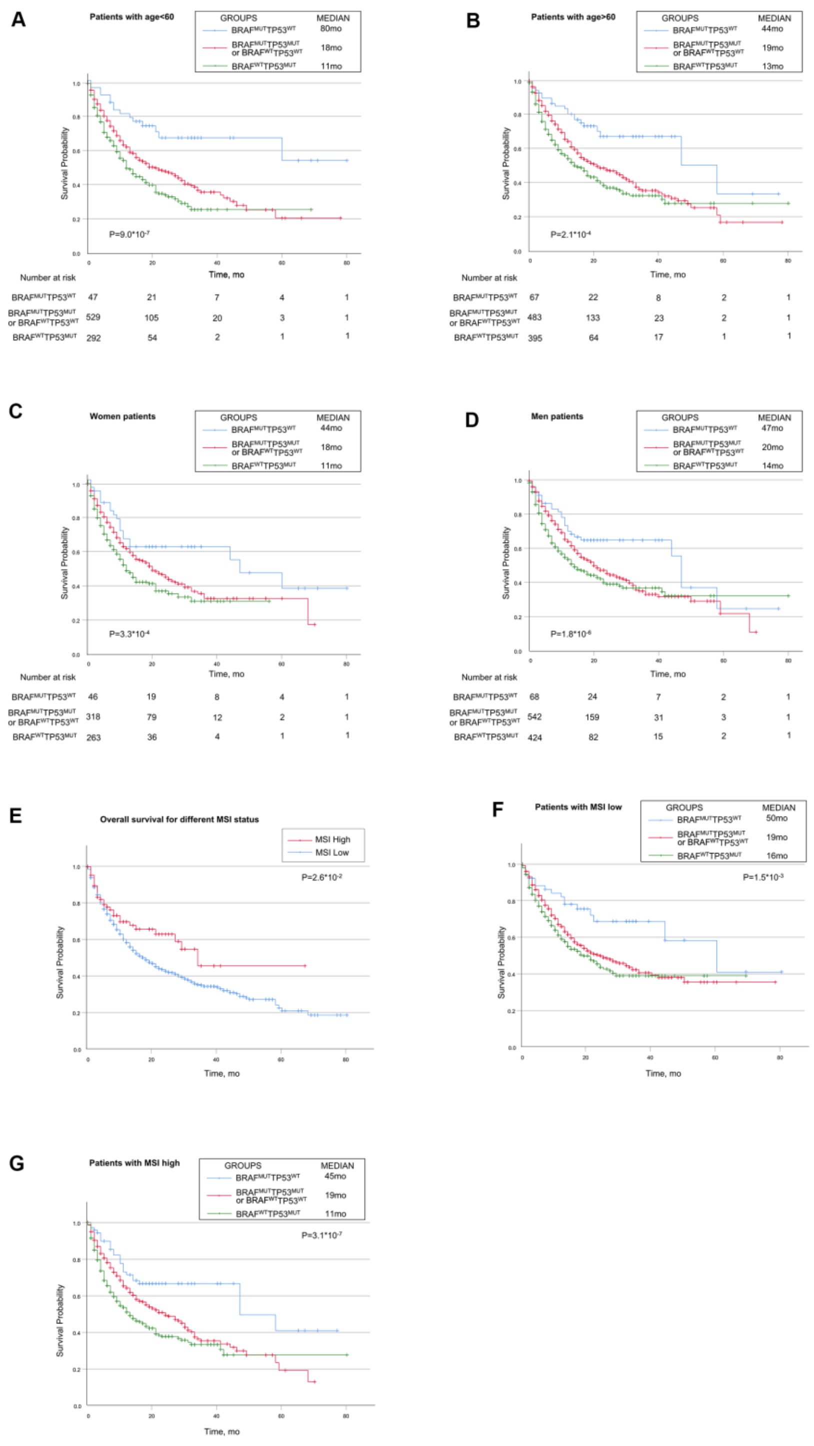

Supplementary Figure 1. Relationship between TP53 and BRAF Mutation Types and Prognosis in Patients Treated with Immune Checkpoint Inhibitors in Different Stratification of Clinical Parameters (**A**, **B**) Overall survival of different TP53/BRAF mutation types in different age groups (age<60 and >60). (**C**, **D**) Overall survival of different TP53/BRAF mutation types in different gender groups (women and men). (**E**) Patients in high-MSI status group had longer OS than patients in low-MSI status group. (**F**, **G**) Overall survival of different TP53/BRAF mutation types in high/low MSI groups.

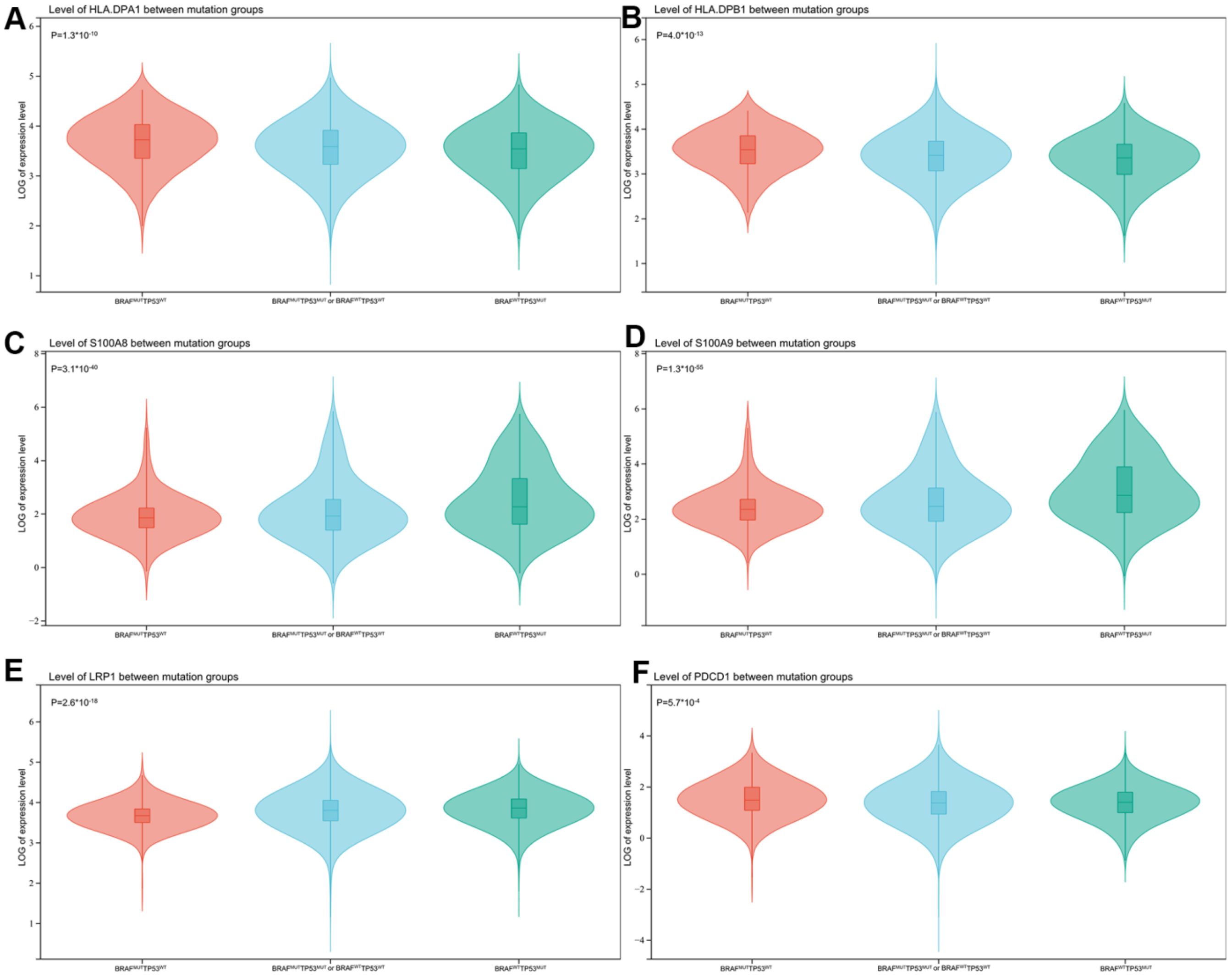

Supplementary Figure 2. Associations of TP53 and BRAF Mutation Types and Immune-related Genes (**A**–**F**) TP53/BRAF mutation model was significantly related to the expression of immune-related genes, that include myeloid-derived suppressor cells (MDSC), major histocompatibility complex (MHC), and immune checkpoints related genes.
